# Cultivar Mixture Cropping Increased Water Use Efficiency in Winter Wheat under Limited Irrigation Conditions

**DOI:** 10.1371/journal.pone.0158439

**Published:** 2016-06-30

**Authors:** Yunqi Wang, Yinghua Zhang, Wei Ji, Peng Yu, Bin Wang, Jinpeng Li, Meikun Han, Xuexin Xu, Zhimin Wang

**Affiliations:** College of Agronomy, China Agricultural University, Beijing, 100193, China; Institute of Genetics and Developmental Biology, CHINA

## Abstract

The effects of cultivar mixture cropping on yield, biomass, and water use efficiency (WUE) in winter wheat (Triticum aestivum L.) were investigated under non-irrigation (W0, no irrigation during growth stage), one time irrigation (W1, irrigation applied at stem elongation) and two times irrigation (W2, irrigation applied at stem elongation and anthesis) conditions. Nearly 90% of cultivar mixture cropping treatments experienced an increase in grain yield as compared with the mean of the pure stands under W0, those for W1 and W2 were 80% and 85%, respectively. Over 75% of cultivar mixture cropping treatments got greater biomass than the mean of the pure stands under the three irrigation conditions. Cultivar mixture cropping cost more water than pure stands under W0 and W1, whereas the water consumption under W2 decreased by 5.9%–6.8% as compared with pure stands. Approximately 90% of cultivar mixtures showed an increase of 5.4%–34.5% in WUE as compared with the mean of the pure stands, and about 75% of cultivar mixtures had 0.8%–28.5% higher WUE than the better pure stands under W0. Similarly, there were a majority of mixture cropping treatments with higher WUE than the mean and the better one of the pure stands under W1 and W2. On the whole, proper cultivar mixture cropping could increase yield and WUE, and a higher increase in WUE occurred under limited irrigation condition.

## Introduction

Wheat (Triticum aestivum L.) is a major grain crop in China [1], and the demand for wheat supply will increase in the following decades [2]. The Huang-Huai-Hai Plain is a main winter wheat producing area in China. However, in this plain, approximately 70%–80% of annual rainfall concentrates in summer maize season, whereas only 20%–30% falls in winter wheat season, which can only meet 25%–40% of requirement in winter wheat, leading to a deficit for 200–300 mm water [3, 4]. A supplementary irrigation of more than 400 mm water was applied, carried out three to four times per season, achieving high grain yield of wheat [5]. However, overdraft of groundwater has resulted in a rapid decline in the groundwater table, threatening sustainable agricultural development in the region [5, 6]. To stabilize the groundwater table, it is urgent to explore a minimum-irrigation strategy that maintain yield and further increase water use efficiency (WUE).

During the past century, genetic improvements have evidently enhanced grain yield of wheat in China [7–11]. Much of the genetic gain in wheat yield has been attributed to increased stress tolerance [12–14]. Additionally, there is an increasing consensus that diversity in functional traits offers a mechanistic bridge between diversity and productivity [15, 16]. As water resources for agronomic use become more limiting, cultivars with different resistance or stress tolerance are sown in mixture cropping system, which maybe a viable solution for maintaining sustainable winter wheat production under limited water conditions.

Cultivar mixtures are mixtures of cultivated cultivars growing simultaneously on the same field with no attempt to breed for phenotypic uniformity [17]. An advantage of cultivar mixtures has been demonstrated especially in terms of containment of fungal diseases [18–20]. Meanwhile, a yield advantage of mixed cultivars has also been observed in various crops including maize [21], barley [22–24], soybean [25] and rice [19]. Of course, many negative effects have also been reported, and often both positive and negative mixing effects have been observed in the same area [21]. For instance, there was no advantage in oat mixtures [26] and barley cultivars mixtures [27]. Previous results were mainly from short time studies. A long term research may gain reliable results. In addition, there is little information on the effect of cultivar mixture on soil water consumption and WUE.

We hypothesized that cultivar mixture cropping can affect population matter production and water consumption characteristics and finally improve yield and WUE by biodiversity and compensatory effect. Therefore, a four years study was carried out to clarify the effect of cultivar mixtures on the yield, biomass, water consumption (ET) and water use efficiency with different cultivars and mixture ratios under limited irrigation conditions.

## Materials and Methods

### Experimental field and meteorological conditions

**Fig 1:**
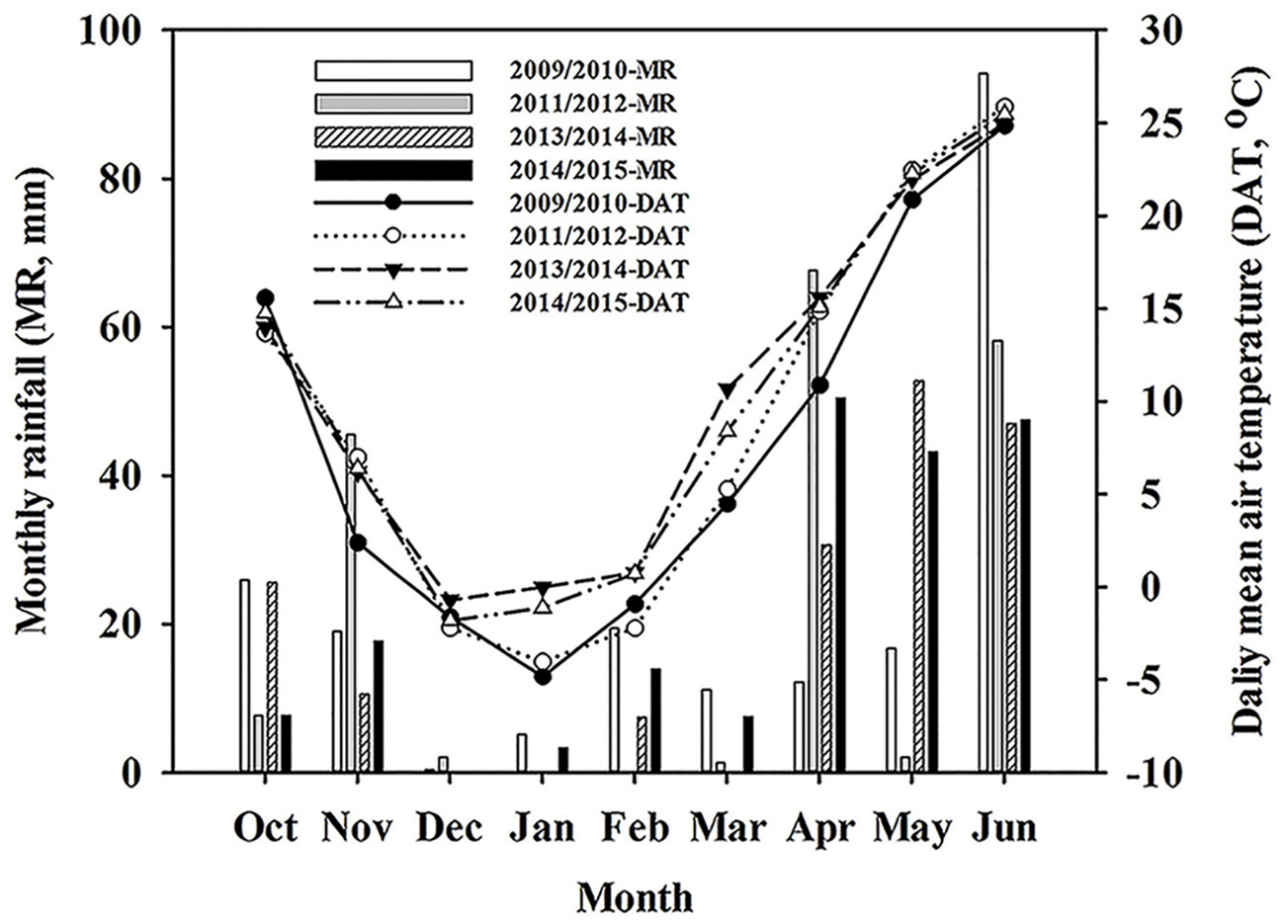
Rainfall and air temperature in the four growing seasons.

Winter wheat experiments were conducted at Wuqiao Experimental Station of China Agricultural University (37°41N, 116°37E, and 18 m above sea level) at Cangzhou, Hebei province, China, in 2009/2010, 2011/2012, 2013/2014 and 2014/2015. No specific permissions were required in the experimental site. The field studies did not involve endangered or protected species. The region has a temperate continental monsoon climate. Total annual illumination is 2724.8 h, with an average temperature of 12.9°C. Average frost free growing days are 201 days, with annual total precipitation amounts of 562 mm. The 64% of the annual rainfall fell in the summer months from July to September. The underground water table was 6–9 m. Maximum water storage was 640 mm, and available water storage was 420 mm in the upper 200 cm soil layer. Soil moisture was 21.7% at maximum field capacity. The wilting coefficient was 7.6%. Soil was clay-loam with an average bulk density of 1.5 g cm^−3^ in the upper 100 cm layer. Climatic data was given in Fig 1. During wheat growing season, total precipitation was 111.7 mm in 2009/2010, 147.9 mm in 2011/2012, 132.1 mm in 2013/2014, and 182.2 mm in 2014/2015. Averaged temperatures in 2009/2010, 2011/2012, 2013/2014 and 2014/2015 growing seasons were 7.0°C, 8.6°C, 9.7°C, and 9.4°C, respectively.

### Plant materials and experimental design

Seven winter wheat cultivars were used in this work. They were Xingmai 4 (X: about 80 cm plant height, stronger tillering ability, half compact plant type and middle spike), Shimai 15 (S: about 75 cm plant height, stronger tillering ability, compact plant type and small spike), Jimai 22 (J: about 75 cm plant height, middle tillering ability, compact plant type and middle spike), Hengguan 35 (G: about 77 cm plant height, middle tillering ability, loose plant type and big spike), Weimai 8 (W: about 85 cm plant height, weak tillering ability, compact plant type and big spike), B 13 (B: about 110 cm plant height, weak tillering ability, loose plant type and big spike), Nongda 399 (N: about 68 cm plant height, stronger tillering ability, compact plant type and middle spike).

The field experiments were designed as split˗plot experiments with irrigation pattern as the main plot and planting pattern as the sub-plot, with four replicates. The plot size was 6m×10 m. There were three irrigation patterns during the whole growth stage (W0: no irrigation during the whole growth stage; W1: irrigation at stem elongation; W2: irrigation at stem elongation and anthesis) and three cultivar mixture ratios (1:1, 1:2, 1:3). The details were listed in Table 1. Grain weight and grain volume of all the cultivars were investigated, and seeds from different cultivars were mixed by seed numbers according to mixture ratio before sowing. The pure stands were planted as control. All experiments received 225 kg N ha^−1^ (as urea), 300 kg P ha^−1^ (as ammonium monoacid phosphate), 150 kg K ha^−1^ (as potassium sulfate), and 75.0 mm irrigation water before sowing. No fertilizer was applied during growing season. Winter wheat was sown with a row space of 15 cm, and the seed density was 4.5×10^6^ seeds ha^-1^.

**Table 1:** Experimental treatments.

Year	Sowing date	Irrigation pattern	Planting pattern	Mixture ratio
2009/2010	October 12, 2009	W1: 75 mm applied at stem elongation; W2: 75 mm applied at stem elongation and 75 mm applied at anthesis.	SG1:2	1:2
			SG1:3	1:3
			SJ1:2	1:2
			SJ1:3	1:3
			XJ1:2	1:2
			XJ1:3	1:3
			S	/
			G	/
			J	/
			X	/
2011/2012	October 14, 2011	W1: 75 mm applied at stem elongation; W2: 75 mm applied at stem elongation and 75 mm applied at anthesis.	SJ1:1	1:1
			SJ1:2	1:2
			SJ1:3	1:3
			WJ1:1	1:1
			WJ1:2	1:2
			WJ1:3	1:3
			S	/
			J	/
			W	/
2013/2014	October 15, 2013	W0: no irrigation during the whole growth stage; W1: 75 mm applied at stem elongation.	BN1:2	1:2
			BN1:3	1:3
			BJ1:2	1:2
			BJ1:3	1:3
			WN1:2	1:2
			WN1:3	1:3
			WJ1:2	1:2
			WJ1:3	1:3
			SN1:2	1:2
			SN1:3	1:3
			B	/
			N	/
			J	/
			W	/
			S	/
2014/2015	October 15, 2014	W0: no irrigation during the whole growth stage; W1: 75.0 mm applied at stem elongation.	BN1:1	1:1
			BN1:2	1:2
			BN1:3	1:3
			SN1:1	1:1
			SN1:2	1:2
			SN1:3	1:3
			B	/
			N	/
			S	/

### Sample measurements

Soil water content (%) was determined gravimetrically at pre-sowing and harvest. Soil samples were taken from 0 to 200 cm in layer segments of 20 cm by using a ground auger, and dried at 105°C to constant weight. Soil water content in each layer was recorded as soil percent moisture content and bulk density. The total soil water consumption (ET) during the whole season was calculated according to water balance equation [28] as below:

ET = P+I+ΔSWS−R−D+CR

Where ET is the total soil water consumption (include evaporation and transpiration); P and I are the rainfall and irrigation quota, respectively; R is the surface runoff; D is the water leakage of plough layer; ΔSWS is the soil water depletion from sowing to maturity; CR is capillary rise into the root zone. Because the groundwater table at the experimental site is 7–9 m (> 4 m) below the ground surface, CR is negligible. R and D can also be ignored in this site.

All plants above ground from four 50 cm site (avoid border rows) in each plot were sampled at maturity, and oven-dried at 70°C until constant weight, and then biomass were measured. In each plot, all plants from two 5 m^2^ site (avoiding border rows) were harvested at dead ripe stage for the determination of grain yield. Actual yield was adjusted by the grain water content of 13%. Water use efficiency (WUE, ratio of grain yield to ET) were calculated.

To describe the differences of yield, biomass, and WUE between cultivar mixture and pure stands, mid-parent superiority (MS, %) and better-parent superiority (BS, %) were calculated for each mixture cropping treatment in reference to the method [29] as below:

$$\text{MS }=\;\frac{\text{S}-\text{PM}}{\text{PM}}\times 100\text{\%}$$

$$\text{BS }=\;\frac{\text{S}-\text{PB}}{\text{PB}}\times 100\text{\%}$$

Where S is the yield, biomass, or WUE of a cultivar mixture cropping treatment, PM is the mean of yield, biomass, or WUE of the component cultivar in pure stands, and PB is the highest yield, biomass, or WUE of the component cultivar in pure stands.

### Statistical analysis

The statistical analysis was performed with the Statistical Analysis System software package [30]. The data of wheat yield, biomass and water use efficiency were subjected to Analysis of Variance using Proc GLM and orthogonal contrast analyses of linear, quadratic and residual effects for quantitative treatments. Duncan’s multiple range tests was used to compare mean differences among treatments at the 5% probability level.

## Result

**Fig 2:**
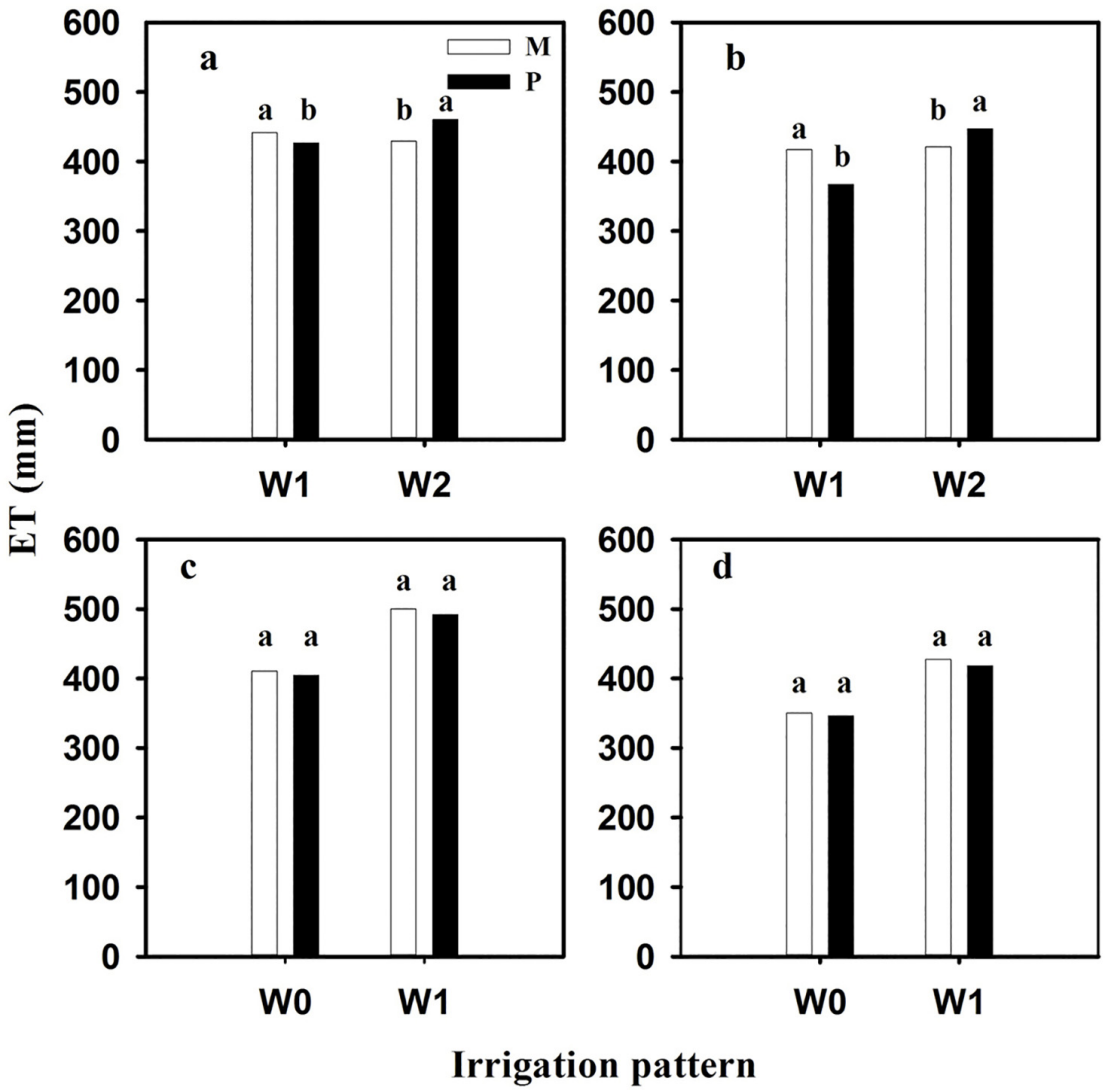
Average ET of winter wheat in four growing seasons. Values indicate means of cultivar mixture cropping treatments or pure stands in 2009/2010 (a; mixture treatments: n = 24; pure stands: n = 16), 2011/2012 (b; mixture treatments: n = 24; pure stands: n = 12), 2013/2014 (c; mixture treatments: n = 40; pure stands: n = 20), and 2014/2015 (d; mixture treatments: n = 24; pure stands: n = 12). M: cultivar mixture treatment; P: pure stands; W0: non-irrigation during the whole growth stage; W1: irrigation at stem elongation; W2: irrigation both at stem elongation and anthesis. Different letters in the same irrigation pattern mean significant difference at 5% level between planting patterns under each year.

Irrigation, planting pattern and their interaction significantly affected yield, biomass and water use efficiency (WUE) in the four growing seasons (Tables 2–5). In 2009/2010, under one time irrigation (W1), the yield of cultivar mixture cropping treatments ranged from 7167.4 kg ha^−1^ in XJ1:3 to 7785.4 kg ha^−1^ in SJ1:2, with positive mid-parent superiority and better-parent superiority (Table 2). XJ1:2 had the greatest biomass with 17570.7 kg ha^−1^, while SG1:3 got the lowest biomass with 15624.6 kg ha^-1^, and all the cultivar mixture treatments had positive mid-parent superiority and better-parent superiority (except SG1:2 and SG1:3). The water consumption was higher in cultivar mixture treatments than in control (Fig 2a). WUE increased from 1.55 kg m^−3^ ha^−1^ in XJ1:3 to 1.79 kg m^−3^ ha^−1^ in SJ1:2, with positive mid-parent superiority (except XJ1:3) and better-parent superiority (except XJ1:3) under W1 (Table 2). Under two times irrigation (W2), the yield of cultivar mixture treatments ranged from 7327.6 kg ha^−1^ in XJ1:2 to 8489.2 kg ha^-1^ in SJ1:3, with positive mid-parent superiority (except XJ1:2 and SG1:3) and better-parent superiority (except SG1:2, XJ1:2 and SG1:3) (Table 2). SJ1:3 had the highest biomass, whilst SG1:2 had the lowest one, and all the treatments had positive mid-parent superiority (except SG1:2) and better-parent superiority (except SG1:2 and XJ1:3). ET in cultivar mixture treatment was lower significantly than pure stands (Fig 2a). WUE in cultivar mixture treatments ranged from 1.70 kg m^−3^ to 2.0 kg m^−3^, with positive mid-parent superiority and better-parent superiority (except XJ1:2) (Table 2). Mean yield, biomass, WUE, and the mid-parent superiority and better-parent superiority of WUE were higher in W2 than in W1, while the mid-parent superiority and better-parent superiority of yield and biomass were higher in W1 than in W2.

**Table 2:** Yield, biomass and water use efficiency (WUE) of cultivar mixture cropping treatments and pure stands of winter wheat as well as the mid-parent superiority (MS) and better-parent superiority (BS) of mixture stands under one time irrigation (W1) and two times irrigation (W2) conditions in 2009/2010.

Irrigation pattern	Planting pattern	Yield			Biomass			WUE		
(I)	(P)	Yield	MS	BS	Biomass	MS	BS	WUE	MS	BS
		(kg ha^-1^)	(%)	(%)	(kg ha^-1^)	(%)	(%)	(kg m^-3^)	(%)	(%)
W1	SG1:2	7534.6b	12.6a	12.5a	16002.6bc	6.5d	-1.9e	1.68b	3.9d	1.2d
	SG1:3	7267.2c	12.9a	12.7a	15624.6c	3.9e	-4.2f	1.67b	2.8e	0.2e
	SJ1:2	7785.4a	13.0a	9.7b	17444.7a	14.5b	7.0c	1.79a	13.5a	13.5a
	SJ1:3	7553.4b	4.0c	1.0d	16589.0b	8.9c	1.7d	1.72b	8.6b	8.6b
	XJ1:2	7233.8c	4.9c	1.9d	17570.7a	23.5a	23.0a	1.69b	6.4c	7.1c
	XJ1:3	7167.4c	7.7b	4.6c	17267.9a	21.4ab	20.8b	1.55c	-2.4f	-1.7f
	S	6680.4d	/	/	16309.9b	/	/	1.58c	/	/
	G	6700.0d	/	/	13753.7e	/	/	1.66b	/	/
	J	7097.9cd	/	/	14162.3d	/	/	1.58c	/	/
	X	6690.9d	/	/	14290.1d	/	/	1.60bc	/	/
	Mean	7171.1	9.2	7.1	15901.6	13.1	7.7	1.65	5.5	4.8
W2	SG1:2	8007.8c	1.0d	-1.5d	16811.2d	-1.5e	-6.2d	1.81bc	3.9c	1.6c
	SG1:3	7763.1d	-2.1e	-4.5e	18809.5bc	10.2b	4.9b	1.80c	3.4d	1.1d
	SJ1:2	8247.2b	2.9c	1.4c	18515.1c	1.8c	0.4c	1.99a	13.0ab	14.5ab
	SJ1:3	8489.2a	5.9a	4.4a	20571.7a	13.1a	11.6a	2.00a	13.4a	14.9a
	XJ1:2	7327.6e	-5.1f	-7.3f	19355.7b	11.7ab	5.0b	1.70d	2.4e	-2.0e
	XJ1:3	8133.7bc	5.3ab	3.0b	17360.3d	0.2d	-5.8d	1.87b	12.4b	7.6b
	S	8130.2bc	/	/	17928.3d	/	/	1.78c	/	/
	G	7724.2d	/	/	16197.2e	/	/	1.71d	/	/
	J	7900.5c	/	/	18436.9c	/	/	1.74d	/	/
	X	7546.6de	/	/	16222.0e	/	/	1.59e	/	/
	Mean	7927.0	1.3	-0.8	18020.8	5.9	1.7	1.8	8.1	6.3
F value	I	****	****	****	***	****	****	***	****	****
	P	**	****	****	****	****	****	***	****	****
	I*P	****	****	****	****	****	****	****	****	****

Different letters within the same column mean significant difference at 5% level among planting patterns under each irrigation condition.

**, *** and **** indicate significance at P < 0.01, P < 0.001 and P < 0.0001, respectively.

**Table 3:** Yield, biomass and water use efficiency (WUE) of cultivar mixture cropping treatments and pure stands of winter wheat as well as the mid-parent superiority (MS) and better-parent superiority (BS) of mixture stands under one time irrigation (W1) and two times irrigation (W2) conditions in 2011/2012.

Irrigation pattern	Planting pattern	Yield			Biomass			WUE		
(I)	(P)	Yield	MS	BS	Biomass	MS	BS	WUE	MS	BS
		(kg ha^-1^)	(%)	(%)	(kg ha^-1^)	(%)	(%)	(kg m^-3^)	(%)	(%)
W1	SJ1:1	7425.4e	5.4e	1.4e	14536.8e	0.2e	-11.5f	1.94c	2.0d	-0.1d
	SJ1:2	7898.2d	12.1d	7.8d	16245.3c	12.0cd	-1.1d	1.94c	2.1d	0.0d
	SJ1:3	8365.9c	18.8c	14.2c	16435.8c	13.3c	0.1c	1.91c	0.9e	-1.3e
	WJ1:1	8112.6cd	17.6c	15.2c	15686.8d	11.6d	-4.5e	2.08ab	18.1b	7.5b
	WJ1:2	8868.6b	28.5b	25.9b	17917.8a	27.5a	9.1a	1.99b	12.8c	2.6c
	WJ1:3	9222.6a	33.6a	31.0a	17111.9b	21.8b	4.2b	2.12a	20.0a	9.2a
	S	7325.2e	/	/	12586.9e	/	/	1.86d	/	/
	J	6760.4g	/	/	16418.5c	/	/	1.94c	/	/
	W	7041.8f	/	/	11683.1f	/	/	1.59e	/	/
	Mean	7891.2	19.3	15.9	15402.5	14.4	-0.6	1.93	9.3	3
W2	SJ1:1	8176.5d	4.8d	-0.9c	16401.8c	6.0e	-5.0e	1.99bc	15.7d	-5.4e
	SJ1:2	8300.4d	6.4d	0.6c	15663.8d	1.3f	-9.3f	1.93c	12.2e	-8.2f
	SJ1:3	8619.3c	10.5c	4.4b	16644.2c	7.6d	-3.6d	2.11ab	22.4c	0.1b
	WJ1:1	8677.4bc	16.0b	5.1b	17282.5b	17.5c	0.1c	2.05abc	22.0c	-2.5d
	WJ1:2	8960.6ab	19.8a	8.6a	17602.6b	19.7b	1.9b	2.16a	28.4a	2.5a
	WJ1:3	9081.2a	21.4a	10.0a	18233.9a	24.0a	5.6a	2.10ab	24.5b	-0.6c
	S	7353.5e	/	/	13665.7e	/	/	1.34d	/	/
	J	8253.4d	/	/	17268.5b	/	/	2.11ab	/	/
	W	6711.1f	/	/	12149.2f	/	/	1.26d	/	/
	Mean	8237.0	13.2	4.6	16101.4	12.7	-1.7	1.89	20.9	-2.4
F value	I	****	****	****	****	****	ns	*	****	****
	P	****	****	****	****	****	ns	***	****	****
	I*P	**	****	****	****	****	ns	*	****	****

Different letters within the same column mean significant difference at 5% level among planting patterns under each irrigation condition. ns: non-significant;

*, **, *** and **** indicate significance at P < 0.05, P < 0.01, P < 0.001 and P < 0.0001, respectively.

**Table 4:** Yield, biomass and water use efficiency (WUE) of cultivar mixture treatments and pure stands of winter wheat as well as the mid-parent superiority (MS) and better-parent superiority (BS) of mixture stands under non-irrigation (W0) and one time irrigation (W1) conditions in 2013/2014.

Irrigation pattern	Planting pattern	Yield			Biomass			WUE		
(I)	(P)	Yield	MS	BS	Biomass	MS	BS	WUE	MS	BS
		(kg ha^-1^)	(%)	(%)	(kg ha^-1^)	(%)	(%)	(kg m^-3^)	(%)	(%)
W0	BN1:2	8228.0e	7.4g	4.0f	20036.4abc	14.0c	8.3a	2.05e	10.3f	10.0e
	BN1:3	8651.4cd	12.9f	9.4d	19541.7cd	11.2d	5.5c	2.07e	11.4f	11.1d
	BJ1:2	8946.1c	9.6f	6.3e	18390.8e	-1.5h	-11.3h	2.13d	11.0f	8.1f
	BJ1:3	9736.8a	19.3d	15.7c	20675.8a	10.7d	-0.2e	2.21c	15.6d	12.6d
	WN1:2	8787.5cd	35.5b	58.2a	16983.9f	-3.2i	2.4d	2.14d	28.6b	15.7c
	WN1:3	8216.8e	9.7f	10.8d	18251.2e	2.9f	-1.4f	2.08e	13.6e	12.1d
	WJ1:2	9682.1a	38.6a	15.0c	20285.1ab	8.8e	-2.1f	2.31b	34.2a	17.6b
	WJ1:3	8204.1e	17.4e	-2.5g	18906.7de	1.4g	-8.8g	2.08e	20.5c	5.6g
	SN1:2	9256.5b	32.5c	24.8b	19422.1cd	16.8b	4.9c	2.38a	34.5a	28.5a
	SN1:3	8544.5d	22.3d	15.2c	19813.0bc	19.2a	7.0b	2.04e	15.0d	9.9e
	B	7908.7ef	/	/	16629.1f	/	/	1.86g	/	/
	N	7414.7f	/	/	18508.7e	/	/	1.85g	/	/
	J	8417.3d	/	/	20727.0a	/	/	1.97f	/	/
	W	5555.9h	/	/	16578.6f	/	/	1.48i	/	/
	S	6561.6g	/	/	14744.1g	/	/	1.69h	/	/
	Mean	8274.1	20.5	15.7	18632.9	8	0.4	2.02	19.5	13.1
W1	BN1:2	9778.8ab	4.7d	1.3d	20036.6cd	5.3d	2.5d	1.87cd	-2.0f	-2.8f
	BN1:3	9512.7bc	1.8e	-1.5e	20832.4b	9.5b	6.5c	1.83e	-4.5h	-5.3h
	BJ1:2	9603.8abc	1.3e	-0.6e	19077.0ef	-0.9f	-2.5e	1.85de	-3.0g	-4.2g
	BJ1:3	8803.1d	-7.1g	-8.8g	20669.5bc	7.3c	5.7c	1.70f	-10.6i	-11.6i
	WN1:2	9659.0abc	12.5a	18.6a	18425.3f	-2.1g	-3.8f	1.85de	-1.8e	-1.4e
	WN1:3	9889.7a	8.4b	9.6b	18448.0f	-0.6g	-0.3de	1.91cd	2.2d	0.5d
	WJ1:2	9379.6c	7.5c	0.9d	19603.6e	2.9e	2.4d	1.98b	5.2c	5.2c
	WJ1:3	9534.8bc	9.3b	2.6d	21505.2a	12.9a	12.3a	2.01b	6.9b	6.9b
	SN1:2	8647.9d	-3.7f	-4.3f	20009.9cd	9.2b	8.1b	1.84de	-1.9f	-3.2f
	SN1:3	9712.2abc	8.1b	7.6c	19726.0de	7.6c	6.6c	2.08a	11.1a	9.6a
	B	9657.3abc	/	/	19556.7e	/	/	1.93c	/	/
	N	9025.4c	/	/	18508.7f	/	/	1.90cd	/	/
	J	9297.3c	/	/	18956.9ef	/	/	1.88cd	/	/
	W	8146.9e	/	/	19147.7ef	/	/	1.88cd	/	/
	S	8943.8d	/	/	18154.5f	/	/	1.85de	/	/
	Mean	9306.2	4.3	2.5	19510.5	5.1	3.8	1.89	0.2	-0.6
F value	I	**	****	****	****	****	****	**	****	****
	P	****	****	****	***	****	****	****	****	****
	I*P	****	****	****	****	****	****	****	****	****

Different letters within the same column mean significant difference at 5% level among planting patterns under each irrigation condition.

**, *** and **** indicate significance at P < 0.01, P < 0.001 and P < 0.0001, respectively.

**Table 5:** Yield, biomass and water use efficiency (WUE) of cultivar mixture treatments and pure stands of winter wheat as well as the mid-parent superiority (MS) and better-parent superiority (BS) of mixture stands under non-irrigation (W0) and one time irrigation (W1) conditions in 2014/2015.

Irrigation pattern	Planting pattern	Yield			Biomass			WUE		
(I)	(P)	Yield	MS	BS	Biomass	MS	BS	WUE	MS	BS
		(kg ha^-1^)	(%)	(%)	(kg ha^-1^)	(%)	(%)	(kg m^-3^)	(%)	(%)
W0	BN1:1	6524.1b	3.1c	-6.2d	16867.2a	8.2a	1.7a	1.95ab	7.0a	-0.6c
	BN1:2	6729.1b	6.4b	-3.3b	14646.5d	-6.0e	-11.7e	1.92b	5.4c	-2.0d
	BN1:3	5402.5d	-14.6f	-22.3f	16274.2b	4.4b	-1.9c	1.50d	-18.0e	-23.8f
	SN1:1	6593.5b	1.1d	-5.2c	15278.1c	-5.4e	-7.9d	1.98a	5.6bc	0.8b
	SN1:2	6255.2c	-4.1e	-10.1e	16459.7ab	1.9d	-0.7c	1.77c	-5.2d	-9.6e
	SN1:3	7344.0a	12.6a	5.6a	16680.5ab	3.3c	0.6b	1.98a	6.0b	1.1a
	B	5695.8d	/	/	14590.2d	/	/	1.68d	/	/
	N	6955.9ab	/	/	16581.2ab	/	/	1.96ab	/	/
	S	6084.6c	/	/	15726.0b	/	/	1.78c	/	/
	Mean	6398.3	0.8	-6.9	15900.4	1.1	-3.3	1.84	0.1	-5.7
W1	BN1:1	8610.4a	18.0a	-4.1a	19084.3a	6.6a	4.2a	2.05a	17.6a	-2.1a
	BN1:2	7864.3b	7.7b	-12.5d	17729.9bc	-1.0e	-3.2f	1.94b	11.6b	-7.2b
	BN1:3	7284.4c	-0.2c	-18.9e	18200.6b	1.6d	-0.6e	1.65d	-5.2c	-21.2e
	SN1:1	8503.4a	-2.0d	-5.3b	17906.1bc	2.8b	2.3b	1.89c	-8.0e	-9.8d
	SN1:2	8332.4a	-3.9e	-7.2c	17745.9bc	1.9d	1.4d	1.92bc	-6.2d	-8.1c
	SN1:3	7949.6b	-8.4f	-11.5d	17805.4bc	2.2c	1.7c	1.92bc	-6.4d	-8.2c
	B	5614.4d	/	/	18315.0b	/	/	1.39e	/	/
	N	8982.9a	/	/	17505.2c	/	/	2.09a	/	/
	S	8365.0a	/	/	17336.6c	/	/	2.01ab	/	/
	Mean	7945.2	1.9	-9.9	17958.8	2.4	1	1.87	0.6	-9.4
F value	I	****	****	****	**	****	****	****	***	****
	P	***	****	****	****	****	****	***	****	****
	I*P	****	****	****	***	***	****	****	****	****

Different letters within the same column mean significant difference at 5% level among planting patterns under each irrigation condition.

**, *** and **** indicate significance at P < 0.01, P < 0.001 and P < 0.0001, respectively.

In 2011/2012, the yield of cultivar mixture cropping treatments ranged from 7425.4 kg ha^-1^ to 9222.6 kg ha^-1^, with positive mid-parent superiority and better-parent superiority under W1 (Table 3). WJ1:2 had the highest biomass, whereas SJ1:1 got the lowest one. All the treatments had positive mid-parent superiority of biomass, but only a half of treatments had positive better-parent superiority. ET in cultivar mixture treatment experienced an increase of 13.8% as compared to pure stands (Fig 2b). WUE varied from 1.91 kg m^−3^ to 2.12 kg m^−3^ in cultivar mixture treatments, with positive mid-parent superiority. Better-parent superiority of WUE in SJ1:1 and SJ1:3 showed negative values, while others showed positive values (Table 3). Under W2 condition, the yield of cultivar mixture treatments ranged from 8176.5 kg ha^−1^ to 9081.2 kg ha^−1^, with positive mid-parent superiority and better-parent superiority (except SJ1:1) (Table 3). WJ1:3 had the greatest biomass, whereas SJ1:2 had the lowest one. All the cultivar mixture treatments got positive mid-parent superiority of biomass, and only WJ1:1, WJ1:2 and WJ1:3 had positive better-parent superiority. ET in cultivar mixture treatment experienced a fall of 5.9% as compared to pure stands (Fig 2b). WUE increased from 1.93 kg m^−3^ in SJ1:2 to 2.16 kg m^−3^ in WJ1:2, with positive mid-parent superiority but negative better-parent superiority (except SJ1:3 and WJ1:2) (Table 3). Mean yield, biomass, and the mid-parent superiority of WUE were higher in W2 than in W1, whilst WUE and the mid-parent superiority and better-parent superiority of yield and biomass were higher in W1 than in W2.

In 2013/2014, the yield ranged from 8204.1 kg ha^-1^ to 9736.8 kg ha^-1^ in cultivar mixture cropping treatments, with positive mid-parent superiority and better-parent superiority (except WJ1:3) under W0 (Table 4). Most of mixture cropping treatments had positive mid-parent superiority for biomass, but only half of the treatments had positive better-parent superiority (Table 4). Cultivar mixture treatment consumed more 6.19 mm water than pure stands, but there was no significant difference between the two treatments (Fig 2c). WUE increased from 2.04 kg m^−3^ to 2.38 kg m^−3^, with positive mid-parent superiority and better-parent superiority (Table 4). Under W1 condition, the yield of mixture cropping treatments increased from 8647.9 kg ha^−1^ to 9889.7 kg ha^−1^, and only two treatments in ten treatments had negative mid-parent superiority, and only four treatments in ten treatments had negative better-parent superiority, others had positive mid-parent superiority and better-parent superiority (Table 4). Biomass of mixture cropping treatments ranged from 18425.3 kg ha^-1^ to 21505.2 kg ha^-1^. BJ1:2, WN1:2 and WN1:3 had negative mid-parent superiority and better-parent superiority, whereas others had positive mid-parent superiority and better-parent superiority (Table 4). Cultivar mixture treatment consumed more 8.43 mm water than pure stands but there was no significant difference between the two treatments (Fig 2c). WUE varied from 1.70 kg m^−3^ to 2.08 kg m^−3^, four treatments had positive mid-parent superiority and better-parent superiority under W1 (Table 4). Mean yield, biomass, and the better-parent superiority of biomass were higher in W1 than in W0, while WUE, the mid-parent superiority of yield, biomass and WUE, and the better-parent superiority of yield and WUE were higher in W0 than in W1.

In 2014/2015, the yield ranged from 5402.5 kg ha^−1^ to 7344.0 kg ha^−1^ in cultivar mixture treatments under W0. Most of the mixture treatments got positive mid-parent superiority but negative better-parent superiority (Table 5). Biomass of mixture cropping treatments ranged from 14646.5 kg ha^−1^ to 16867.2 kg ha^−1^, and four in six treatments had positive mid-parent superiority but negative better-parent superiority (Table 5). Cultivar mixture treatment consumed more 3.88 mm water than pure stands, but there was no significant difference between the two treatments (Fig 2d). WUE varied from 1.50 kg m^−3^ to 1.98 kg m^−3^, two-thirds of treatments had positive mid-parent superiority of WUE, only two treatments got positive better-parent superiority (Table 5). Under W1 condition, the yield of mixture cropping treatments ranged from 7284.4 kg ha^−1^ to 8610.4 kg ha^−1^, only two treatments got positive mid-parent superiority, and all the treatments got negative better-parent superiority. Biomass of mixture cropping treatments increased from 17729.9 kg ha^−1^ to 19084.3 kg ha^−1^, with positive mid-parent superiority (except BN1:2) and better-parent superiority (except BN1:2 and BN1:3) (Table 5). Cultivar mixture treatment consumed more 9.55 mm water than pure stand, but there was no significant difference between the two treatments (Fig 2d). WUE of mixture cropping treatments varied from 1.65 kg m^−3^ to 2.05 kg m^−3^, and most treatments had negative mid-parent superiority and better-parent superiority under W1 (Table 5). Mean yield, biomass, WUE, and their mid-parent superiority were higher in W1 than in W0, while most treatments of the better-parent superiority were negative under the two irrigation treatments.

**Fig 3:**
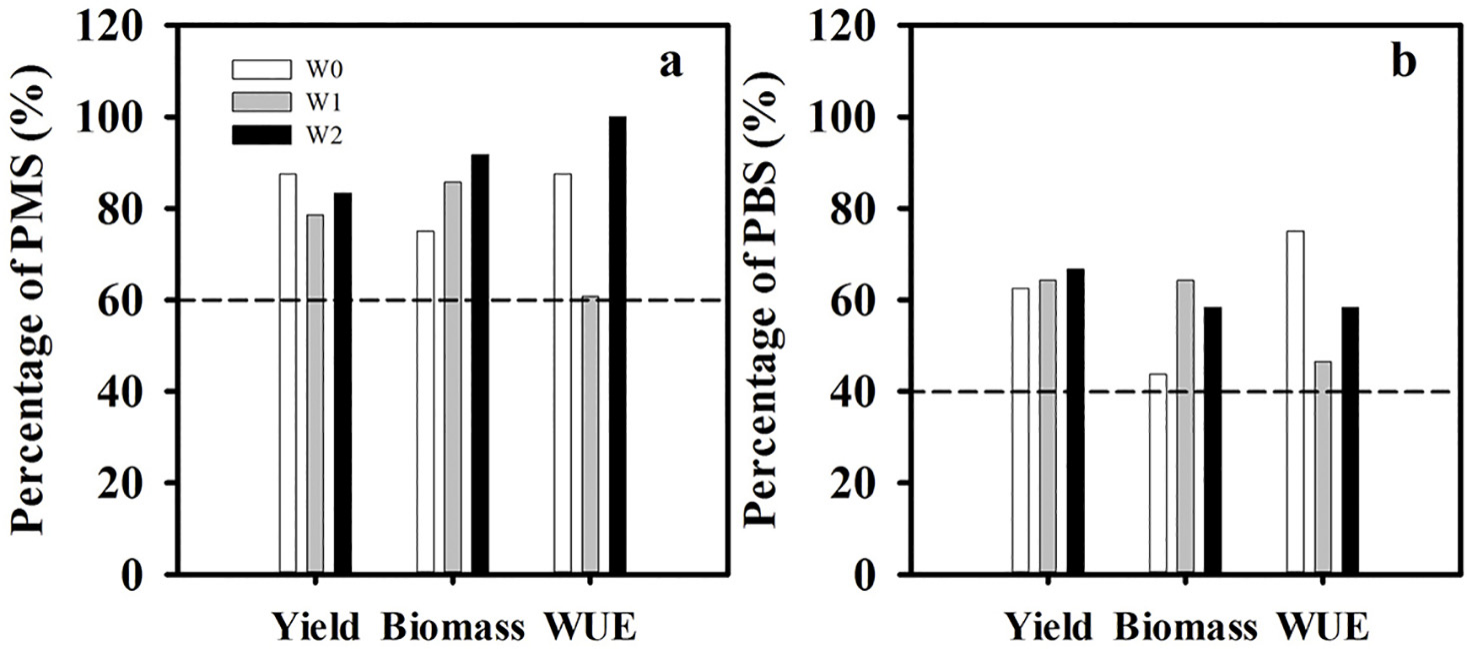
Percentage of positive mid-parent superiority (PMS, a) and positive better-parent superiority (PBS, b) for yield, biomass and water use efficiency (WUE). Values indicate means of samples for each irrigation treatment (W0: n = 64; W1: n = 112; W2: n = 64). W0: non-irrigation during the whole growth stage; W1: irrigation at stem elongation; W2: irrigation both at stem elongation and anthesis.

Comprehensive data of 4 years showed that over 60% cultivar mixture treatments had positive mid-parent superiority of yield, biomass, and WUE (Fig 3a), while the figure for better-parent superiority stood at more than 40% (Fig 3b). The percentage of positive mid-parent superiority of yield under W0, W1 and W2 was around 90%, nearly 80% and about 85% (Fig 3a), respectively, whereas the figure for better-parent superiority was just over 60% (Fig 3b). The percentage of positive mid-parent superiority of biomass increased from W0, W1, to W2 (Fig 3a). The percentage for positive better-parent superiority of biomass was highest in W1, and it was lowest in W0 (Fig 3b). The percentage for positive mid-parent superiority of WUE was highest in W2, and it was lowest in W1 (Fig 3a). The percentage of positive better-parent superiority of WUE was highest in W0, and it was lowest in W1 (Fig 3b). In addition, although there was weak difference in yield, biomass and WUE of the same cultivar mixture patterns among different years, they had similar mid-parent and better-parent superiority. For example, SJ1:2 gained yield of 7785.4 kg ha^-1^, biomass of 17444.7 kg ha^−1^ and WUE of 1.79 kg m^−3^ under W1 in 2009/2010 (Table 2), whilst the figure was 7898.2 kg ha^−1^, 16245.3 kg ha^−1^ and 1.94 kg m^−3^ in 2011/2012 (Table 2), and there was positive mid-parent and better-parent superiority in two growing seasons (Tables 2 and 3). These results showed that the mixture superiority was stable among years.

## Discussion

There are many reports on effects of cultivar mixtures on yield. Some researchers found that there were rises of yield in cultivar mixture cropping systems [31–33], whereas other researchers reported that there was no change in grain yield between mixtures and pure stands [27, 34]. These above reports were based on short term experiments, their results might need to be tested further in long term field experiment. This research selected different plant height, tillering ability, spike-type cultivars, conducted a four growing seasons study under different irrigation amount, and found that well over 80% of all the cultivar mixtures showed a significant growth of yield as compared with mean yield of pure stands, what was more, the figure reached at about 90% under non-irrigation condition (Fig 3a). This was consistent with the prediction that mixtures would have less interaction with the environment than their components on condition that the components had difference in their responses to environment [35]. In addition, cultivars mixtures could make full use of different space-time resources, with different agronomic factors (plant height, disease and insect resistance) and higher biodiversity than pure stands, and as result of a marked increase of yield [36–40]. Furthermore, this study found that over 75% of cultivar mixtures got significantly greater biomass than the mean of pure stands. The biomass is controlled by amount of solar radiation absorption that begins during early vegetative growth and continues to physiological maturity [41]. Mixtures of the two wheat cultivars created a wavy type canopy consisted of shorter and taller plants, in contrast to the monoculture of either cultivar, this canopy architecture could make more non-uniform distribution of leaves in group, and had a greater potential for intercepting radiation, producing more dry matter [42]. Additionally, the canopy of the mixtures would have resulted in earlier canopy closure which aided in improving light interception efficiency and crop productivity [43].

In Huang-Huai-Hai Plain, winter wheat grows in season with little rainfall, and thus it is important to increase WUE. This study showed that well over 85% and more than 60% of mixture treatments had higher WUE than the mean of pure stands under non-irrigation and one-time irrigation conditions (Fig 3a). There was no significant difference in ET between mixture cropping and pure stands (Fig 2); however, a large number of mixture treatments had higher yield than pure stands under the two irrigation patterns (Fig 3a). Thus it was concluded that the higher WUE in cultivar mixture cropping as compared with pure stands was due to an evident increase in grain yield under non-irrigation and one-time irrigation conditions, especially under non-irrigation condition. It agreed with a research of mixtures in which the advantage of mixtures was observed in a serious drought season [33] owing to the increasing adaptability of mixtures to buffer plants against unpredictable environmental variation [44, 45]. Some research showed that limited irrigation enhanced root weight in deep soil layer and proportion of green non-leaf area, and water was absorbed from deep soil layer [28], so higher yield and WUE were gained. In this study, almost all the mixture cropping treatments experienced a significant rise of WUE as compared with pure stands under two times irrigation (Tables 2 and 3), which was result of the increase in yield and the reduction in ET. These results might relate with the competition among different cultivars in cultivar mixture cropping system. There were competitions for some resources among different plants or cultivars in the same system. Different plants had various ability of acquiring resources, different crop cultivars held diverse types of root system [33], and thus unbalance competition for resources took place among different plants or cultivars [46, 47]. In other words, there were the strong and the weak competition inter-species or intraspecific in eco-system. In general, the closer the plant ecological niche, the more intense competition, e.g. the flowering stage was the most competitive period for resources [48]. In this study, there was no difference of ecological niche among different winter wheat cultivars, so it was easy to lead to fierce competition for water under limited water condition (W0 and W1), whereas the competition was relieved under two times irrigation condition, according to reduced ET (Fig 2).

In addition, previous researches showed that several different genetic cultivars were sown in the field, resulting in higher resistance and productivity as compared with pure stands [19, 49, 50], and crop heterogeneity is a possible solution to the vulnerability of monocultured crops to disease. In this study, resistance gene in mixture cropping was not measured; however, the leaf area index was higher in mixture population than in pure stands and the senescence was delayed in mixture cropping (data not given).

Cultivar selection and mixture ratio also affected the yield and WUE of mixture cropping. In this study, the average yield of 1:1, 1:2 and 1:3 mixture ratios were 7827.9, 8388.1 and 8352.1 kg ha^-1^, and the highest yield were 8677.4 (WJ1:1), 9778.8 (BN1:2) and 9889.7 (WN1:3) kg ha^-1^, respectively, and there was big difference in plant height, tillering ability, plant type and spike size between the two mixed cultivars for high yield mixture cropping treatments, which could make best use of the complementary effect between cultivars. The average WUE of 1:1, 1:2 and 1:3 mixture ratios were 1.99, 1.94 and 1.91 kg m^-3^, and the highest WUE were 2.08 (WJ1:1), 2.38 (SN1:2) and 2.21 (BJ1:3) kg m^-3^, respectively. It can be seen that the mixture ratios of 1:2 and 1:3 (tall stalk to short stalk) had higher yield and WUE. However, the optimum cultivar match and mixture ratios as well as their effect on population structure, photosynthesis, canopy micro-climate as well as water and nitrogen absorption and utilization need to be further studied.

## Conclusion

The four growing seasons field experiments demonstrated that most mixture cropping treatments had higher water use efficiency (WUE) than pure stands under three irrigated conditions. WUE experienced a significant rise by means of an increase of yield under W0 and W1, while it was improved by an increase in yield and a drop of water consumption under W2. Sum up, cultivar mixtures impressively increased yield and WUE under limited irrigation conditions.

## Supporting Information

S1 Table Monthly rainfall (MP, mm) and daily mean air temperature (AT,°C) in the four growing seasons. (PDF)

S2 Table ET (mm) of winter wheat in four growing seasons. (PDF)

S3 Table Percentage of positive mid-parent superiority (PMS) and positive better-parent superiority (PBS) for yield, biomass and water use efficiency (WUE). (PDF)
