# Supplementary material for: Cultivar Mixture Cropping Increased Water Use Efficiency in Winter Wheat under Limited Irrigation Conditions
Source: PLoS One. 2016 Jun 30;11(6):e0158439. doi: 10.1371/journal.pone.0158439 (PMC4928908; doi:10.1371/journal.pone.0158439)
Supplement: S1 Table — (PDF) [file pone.0158439.s001.pdf]

S1 Table. Monthly rainfall (MP, mm) and daily mean air temperature (AT, ℃) in the four growing seasons.

Month	2009/2010-MP	2011/2012-MP	2013/2014-MP	2014/2015-MP	2009/2010-DAT	2011/2012-DAT	2013/2014-DAT	2014/2015-DAT
Oct	26	7.65	25.7	7.7	15.6	13.7	14	14.8
Nov	19	45.55	10.6	17.7	2.4	7	6.2	6.4
Dec	0.4	2.1	0.1	0	-1.6	-2.2	-0.7	-1.8
Jan	5.2	0	0.1	3.4	-4.8	-4	0	-1.1
Feb	19.5	0	7.4	14	-0.9	-2.2	0.8	0.75
Mar	11.2	1.3	0	7.5	4.5	5.3	10.7	8.4
Apr	12.2	67.7	30.7	50.5	10.9	14.9	15.6	15.1
May	16.8	2.1	52.8	43.2	20.9	22.5	22	22.3
Jun	94.2	58.25	47	47.5	24.9	25.9	25	25.5
