# Supplementary material for: Cultivar Mixture Cropping Increased Water Use Efficiency in Winter Wheat under Limited Irrigation Conditions
Source: PLoS One. 2016 Jun 30;11(6):e0158439. doi: 10.1371/journal.pone.0158439 (PMC4928908; doi:10.1371/journal.pone.0158439)
Supplement: S2 Table — (PDF) [file pone.0158439.s002.pdf]

S2 Table. ET (mm) of winter wheat in four growing seasons.

		average		stdev	
	Irrigation	Mixture	Pure stands	Mixture	Pure stands
2009/2010	W1	441.2	426.7	6.6	8.0
	W2	429.2	460.3	5.8	7.6
2011/2012	W1	417.5	366.8	11.0	5.0
	W2	420.9	447.2	12.0	9.8
2013/2014	W0	410.7	404.5	11.0	6.0
	W1	500.3	491.9	7.8	8.9
2014/2015	W0	350.3	346.4	5.9	6.6
	W1	427.7	418.1	11.0	7.6
