# Supplementary material for: Cultivar Mixture Cropping Increased Water Use Efficiency in Winter Wheat under Limited Irrigation Conditions
Source: PLoS One. 2016 Jun 30;11(6):e0158439. doi: 10.1371/journal.pone.0158439 (PMC4928908; doi:10.1371/journal.pone.0158439)
Supplement: S3 Table — (PDF) [file pone.0158439.s003.pdf]

S3 Table. Percentage of positive mid-parent superiority (PMS) and positive better-parent superiority (PBS) for yield, biomass and water use efficiency (WUE).

Percentage of PMS (%)	W0	W1	W2	Percentage of PBS (%)	W0	W1	W2
Yield	87.5	78.6	83.3	Yield	62.5	64.3	66.7
Biomass	75.0	85.7	91.7	Biomass	43.8	64.3	58.3
WUE	87.5	60.7	100.0	WUE	75.0	46.4	58.3
